# Use of electron backscatter diffraction patterns to determine the crystal lattice. Part 2. Offset corrections

**DOI:** 10.1107/S1600576723000146

**Published:** 2023-02-24

**Authors:** Gert Nolze, Tomasz Tokarski, Łukasz Rychłowski

**Affiliations:** a Federal Institute for Materials Research and Testing (BAM), Unter den Eichen 87, 12205 Berlin, Germany; b Institut für Mineralogie, TU Bergakademie Freiberg, Brennhausgasse 14, 09596 Freiberg, Germany; c Academic Centre for Materials and Nanotechnology, AGH University of Science and Technology, Mickiewicza 30, 30-059 Krakow, Poland; Ecole National Supérieure des Mines, Saint-Etienne, France

**Keywords:** mean atomic number, Kikuchi patterns, lattice parameters, automated Bragg angle determination, lattice parameter determination, dynamical theory of electron diffraction, electron backscatter diffraction, Funk transform

## Abstract

A band width determination using the first derivative of the band profile systematically underestimates the true Bragg angle. Corrections are proposed to compensate for the resulting offset Δ*a*/*a* of the mean lattice parameters derived from as many Kikuchi band widths as possible. For dynamically simulated Kikuchi patterns, Δ*a*/*a* can reach up to 8% for phases with a high mean atomic number *Z*, whereas for much more common low-*Z* materials the offset decreases linearly. A predicted offset Δ*a*/*a* = *f*(*Z*) is therefore proposed, which also includes the unit-cell volume and thus takes into account the packing density of the scatterers in the material. Since *Z* is not always available for unknown phases, its substitution by *Z*_max_, *i.e.* the atomic number of the heaviest element in the compound, is still acceptable for an approximate correction. For simulated Kikuchi patterns the offset-corrected lattice parameter deviation is Δ*a*/*a* < 1.5%. The lattice parameter ratios, and the angles α, β and γ between the basis vectors, are not affected at all.

## Abstract 2

Automatically determined band widths in simulated backscatter Kikuchi patterns exhibit differences from the double Bragg angles that correlate with the scatterer density. Corrections are proposed to compensate for this.

## 1. Introduction

Our systematic investigation of physics-based simulated backscattered Kikuchi diffraction (BKD) patterns in Part I of this series (Nolze *et al.*, 2023 ▸) showed that the Bragg angle in the edge profile of a Kikuchi band is fairly indeterminate. As an alternative, the band width *W*_*hkl*_ = (θ_min_ − θ_max_) as the distance between the (global) extreme positions of the first derivative is proposed as a rough estimate of the double Bragg angle 2θ_*hkl*_. Although the first derivative works automatically and gives reproducible results, it yields slightly different lattice parameters *a*_*hkl*_ from Kikuchi band widths indicating the (inverse) distance to the reciprocal-lattice point *hkl*. The resulting distribution of *a*_*hkl*_ as calculated using the software *CALM* (Nolze *et al.*, 2021 ▸) is described by a mean *a*_*CALM*_ and a standard deviation σ_*hkl*_.

Since for simulated patterns the true lattice parameters are known, the offset Δ*a*/*a* = (*a*_*CALM*_ − *a*_0_)/*a*_0_ can easily be displayed as a function of the mean atomic number 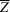 of the respective phase. For simulated patterns, the offset is always positive (0 < Δ*a*/*a* < 8%). To a first approximation it scales with 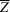 or the backscatter coefficient η that can be derived from it, just as in the study of numerous experimental Kikuchi patterns by Nolze *et al.* (2021 ▸). The lattice parameter offset determined on experimental BKD patterns appears to be shifted by only −4%, such that −4 < Δ*a*/*a* < 4%.

The true reasons for this offset shift are currently unknown. They could be the result of imperfect input values during simulation of the Kikuchi patterns, or arise from systematic errors made in experimental patterns and an incorrectly determined projection centre (PC) or from excess deficiency effects. It is also conceivable that the differences are due to a deviating electron energy, *e.g.* if the effective landing energy is lower than the accelerating voltage. The resulting higher electron wavelength λ_e_ would suggest a shorter translation periodicity of the lattice. Since *a*, *b*, *c* ∝ 1/θ_*hkl*_ ∝ 1/λ_e_ ∝ 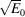 , for *E*_0_ = 20 kV the effective electron energy would have to be about 1.5 keV lower to account for the offset shift of Δ*a*/*a* ≃ 4%.

On the other hand, it is known that not *E*_0_ but rather an energy distribution is likely to be relevant for experimental BKD patterns, whose governing maximum is slightly below *E*_0_ (Wells, 1974 ▸; Reimer, 1998 ▸; Goldstein *et al.*, 2018 ▸). Winkelmann *et al.* (2019 ▸) showed that for Si the mean energy is estimated to be 1–1.5 keV below the discussed *E*_0_ = 15 keV. This agrees surprisingly well with the shift observed here, though this effect is predicted to be progressively smaller for higher- 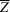 materials. Winkelmann *et al.* (2019 ▸) dealt mainly with the change in the effective electron energy as a function of the scattering angle, which is not considered at all in BKD pattern simulations.

## 2. Offset corrections

If we assume that the offset curve of the simulated BKD patterns also explains that of the experimental patterns to a first approximation, the goal is to predict the offset based on the chemical composition of the phase, for example, and thus be able to correct *a*_*CALM*_.

### 2.1. Elements

For simplicity, the elements are analysed first and then compounds, since for the former 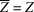 holds.

In Fig. 1 ▸(*a*) the relative offset Δ*a*/*a* is shown as hollow symbols for as many elements as possible of different structure types; for some elements more than a single modification was analysed, *e.g.* for Fe all three are shown. The light-grey error bars refer to σ_*hkl*_ which also increases with *Z*. The distribution of hollow symbols can be roughly described by a linear approach, 

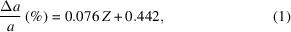

plotted in Fig. 1 ▸(*a*) as a dark-grey line. However, clearly visible hump-shaped deviations from this line occur for elements with *Z* = 25–30, *Z* = 40–50 and *Z* = 70–80.

The positions of these humps are reminiscent of the mass density ρ, shown for comparison in Fig. 1 ▸(*b*) as blue filled circles. The undulating curve is due to the varying binding forces and the resulting similarly varying packing density of the atoms, which can be described in a similar way to the mass density. Therefore, for a better description of the offset curve, the mass density is taken into account, which is as much of an unknown for an unknown phase as the lattice parameters to be determined. However, *a*_*CALM*_ and the unit-cell volume *V*_uc_ derived from it are completely sufficient for a suitable estimate.

The mass density ρ is the quotient of the mass *m*_uc_ and volume *V*_uc_ of the unit cell. *m*_uc_ is equivalent to the number of formula units (*n*) per unit cell multiplied by the atomic mass *M*. Since for elements *M* ≃ 2*Z* we can formulate a relationship between density ρ and atomic number *Z*,

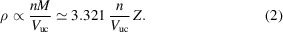

(*n*/*V*_uc_)*Z* represents the proton density, which is assumed to be crucial for backscattering since the interaction happens between the primary electrons and the core of the atom. If there is an anomaly in the proton density, a deviation from the linear lattice parameter offset follows.

Fig. 1 ▸(*b*) proves that, apart from a proportionality factor of 1.25, the density ρ (light-blue circles) for elements is almost congruent with 3.321*nZ*/*V*_uc_ (red filled circles). [The factor 3.321 results from 2 ( ≃ *M*/*Z*) divided by Avogadro’s number 6.02214076 × 10^23^ and multiplied by cm^3^/Å^3^ = 10^24^.]

Accepting the similarity of the hump positions as a sufficient argument for correlation, the offset shown in Fig. 1 ▸(*a*) can be satisfactorily predicted by a quadratic equation with *nZ*/*V*_uc_ as an additional correction term in (1),

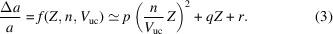

For elements, the factors *p*, *q* and *r* in equation (3) are refined by a least-squares approach to give *p* = 0.098, *q* = 0.064 and *r* = 0.525, which leads to the distribution of black dots shown in Fig. 1 ▸(*a*). By inserting *Z* and *V*_uc_ into (3) the offset can be corrected.

### 2.2. Compounds

If the same correlation between lattice parameter offset and 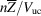 is assumed for compounds, the main challenge is the estimation of the mean atomic number 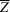 .


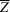
 has been described in the past only in purely empirical terms. A few approaches (I–III) are discussed, for example, by Joy (1995 ▸), Reimer (1998 ▸) and Howell *et al.* (1998 ▸) and use either the mass fraction *c*_*i*_ or the atomic fraction *a*_*i*_:

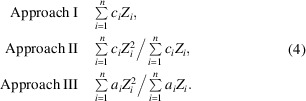

They not only change the wanted (vertical) offset for the phase but also shift the respective point horizontally according to the estimates proposed in (4).

To start with the simplest possible phases, the master patterns of 96 binary phases of type *AB* were simulated and analysed. Among these, there were 43 with structure type *B*1 (NaCl), 12 with *B*2 (CsCl), 16 with *B*3 (zincblende) and eight with *B*4 (wurtzite). Compounds of light, heavy, or both light and heavy elements were selected since we expected the largest deviation in the fit for such phases.

In addition, BKD patterns were analysed for a further 136 common phases that consist of up to five elements and are of greater technical or mineralogical interest, such as borides, carbides, oxides, sulfides, arsenides, selenides, carbonates, sulfates, silicates and various intermetallic phases.

Direct comparisons of the 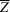 models in (4) show that the smallest discrepancies between the lattice parameter offset from *CALM* and a fit using the quadratic correction in (3) accumulate from approach I: 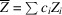 . The best match between the offset result using *CALM* and that predicted using 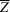 and *V*_uc_ is obtained when the following constants are used in (3): *p* = 0.133, *q* = 0.064 and *r* = 0.686. In Fig. 2 ▸, the predicted offsets are again represented by black dots overlaying the Δ*a*/*a* derived using *CALM* (hollow circles). The different colours of the circles distinguish between the different structure types. For a better comparison with Fig. 1 ▸ the linear fit of (1) is drawn again as a dark-grey straight line.

Fig. 2 ▸ suggests that the quadratic term in equation (3) is not as important for compounds as for elements. The offset of the compounds scatters apparently randomly around the grey line and results in Δ*a*/*a* = ±1%. The distribution of the filled circles confirms that for many phases the difference between linear and quadratic fits is small. Nevertheless, especially for 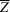 > 60, considerable offsets appear which are well predicted. On the other hand, the majority of binary phases with 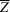 = 70 ± 3 show a lower offset than predicted by the linear or quadratic fits. In addition, the number of compounds is higher than that of elemental structures. The symbols of many phases in Fig. 2 ▸ inevitably overlap or obscure each other, *i.e.* one really only sees the outliers. Comparing the frequency distributions of the relative deviations of the predicted offset (fit) and the offset from *CALM* for elements and compounds, it turns out that they are quite similar (Fig. 3 ▸). They all look similar to a normal distribution. The minimal shift of the maxima of ∼0.1% indicates a slight overestimation of the offset by the applied fit. However, compared with the FWHM of the distribution of ∼0.6% this is negligible.

The difference between the offset determined by *CALM* and the offset predicted for correction should also be related to the uncertainty σ_*hkl*_ in the band width determination, and it characterizes the quality of *a*_*CALM*_. The frequency distributions of |*a*_fit_ − *a*_*CALM*_|/*a*_*CALM*_ and of σ_*hkl*_/*a*_*CALM*_ in Fig. 4 ▸ show that the standard deviation σ_*hkl*_ on *a*_*CALM*_ is considerably higher than imperfect corrections from the fit. This can also be seen in Fig. 2 ▸ when looking at the error bars, which are significantly longer than the deviation of the fitted black points from the corresponding hollow circles.

Considering the larger uncertainty of σ_*hkl*_ in Fig. 4 ▸, the linear approach of equation (1) shown in Fig. 1 ▸ could also be used as-is for a simple correction on *a*_*CALM*_. This simplifies the fitting considerably because one does not need to know *n*, the number of formula units in the unit cell. *V*_uc_, on the other hand, is no problem because, although uncorrected, it is determined sufficiently accurately by *CALM*. However, a remaining problem is that even then the chemical composition of the unknown phase must be known as accurately as possible in order to obtain a reliable 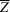 .

#### 2.2.1. Estimation of *n*

The quadratic correction term in (3) contains *n*, the number of formula units distributed in the unit cell, in addition to the unit-cell volume *V*_uc_. For the 249 phases analysed, *n* was of course known. However, this is not the case for an unknown phase.

From equation (3) it follows that Δ*a*/*a* increases with *n*. In Fig. 5 ▸ five phases with different *n* have been chosen as examples. *n* is given as a number in parentheses after each phase in the legend, and is shown in Fig. 5 ▸ for four of the five phases as black filled symbols; the exception is Cu_3_Au, where *n* = 16 is far outside the displayed range.

Since according to (3) the difference between offsets for different *n* is given by 

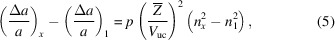

the absolute level at *n* = 1 and the curvatures in Fig. 5 ▸ depend on the phase-specific ratio 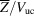 . The curvature increases the higher 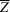 and the lower *V*_uc_. A small *V*_uc_ can only be associated with a small *n*. On the other hand, and as Fig. 5 ▸ shows, for compounds with higher *n* the curvature decreases successively for wrong *n* and has less and less influence on Δ*a*/*a*. Thus, it seems reasonable to assume *n* = 1 for an unknown phase as a first approximation. The resulting deviations between the true *n* and *n* = 1 for all investigated compounds are shown graphically in Fig. 6 ▸ for all analysed phases. Of the 249 compounds considered, 36 have *n* = 1 and mainly represent the first bar in the inset histogram in Fig. 6 ▸. Nearly half the compounds have an offset deviation <0.075%, independent of their true *n*. We conclude that, for unknown phases, the use of *n* = 1 nevertheless leads to acceptable offset corrections. Larger deviations are apparently only to be expected for phases with 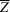 > 60 (Fig. 6 ▸).

The least-squares optimized parameters for offset prediction with *n* = 1 and 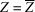 in equation (3) are *p* = 0.113, *q* = 0.069 and *r* = 0.621.

#### 2.2.2. The use of *Z*_max_

The determination of the chemical composition of unknown crystalline phases in a matrix becomes increasingly uncertain, especially in the case of very small inclusions or precipitates, when it cannot be ruled out that a considerable part of the chemical signal used for the determination comes in fact from the surrounding matrix.

However, there is possibly another relationship inherent in 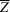 . In approach I in (4) the mass fraction 

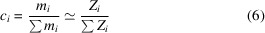

is multiplied by *Z*_*i*_ so that 

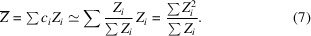

The weighting of heavy elements in the estimation of 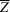 in compounds is clearly higher than that of light elements.

The analysis of all studied compounds showed that the element with the highest atomic number *Z*_max_ can substitute 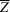 to a good approximation (Fig. 7 ▸): the comparison of 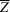 and *Z*_max_ shows an almost proportional relationship (dotted line).

Replacing 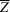 by *Z*_max_ and assuming *n* = 1, the following parameter set can be fitted: *p* = 0.117, *q* = 0.055 and *r* = 0.526. The diagram in Fig. 8 ▸(*a*) displays the predicted offset (black dots) but this time plotted as function of *Z*_max_. Since 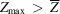 , relative to their position in Fig. 2 ▸ all hollow circles representing the lattice parameter offset derived in *CALM* are shifted to the right, which results in a lower linear slope of the distribution (light-grey dotted line). The remaining offset difference Δ_fit−*CALM*_ between the offset predicted with *Z* = *Z*_max_ and *n* = 1 and the one resulting from *CALM* is shown in Fig. 8 ▸(*b*). Although the deviation is about one-third larger than in Fig. 2 ▸ for the true 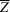 and *n*, it is still often less than 1%, which in our view represents a real alternative for phases whose chemical composition is not well known except for the heaviest element of the compound.

Thus, the resulting correction of the absolute lattice parameters by the predicted offset via *Z*_max_ will not be as good as for a phase with known composition, but we expect that the supposed error increases only for phases with heavy elements and amounts to a maximum of 2% [Fig. 8 ▸(*b*)].

## 3. Conclusions

For simulated Kikuchi patterns *a*_*CALM*_ is systematically too high. The offset approximately correlates with 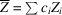 of the phase. However, the use of *Z*_max_ and *n* = 1 is, to a first approximation, also an acceptable approach to estimate the offset Δ*a*/*a*, 

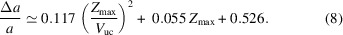

This enables the derivation of a corrected lattice parameter, 

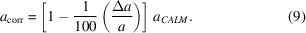

The deviation on the lattice parameter *a*_0_ used during signal simulation of the master pattern is presented in Fig. 9 ▸.

Higher *Z*_max_ tend to generate higher deviations. For the 249 compounds studied, the relative deviation is given by 

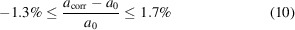

and is therefore clearly smaller than the 10% estimated by Dingley & Wright (2009 ▸). However, these uncertainty limits are only valid for simulated patterns. For experimental patterns additional errors like trace position, projection centre and wavelength distribution will affect the result in such a way that the error will increase.

Since all other lattice parameters, *i.e.* *a*/*b*, *c*/*b*, α, β and γ, remain unchanged, the missing basis vector lengths can be determined: 

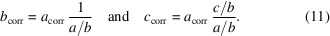




## Figures and Tables

**Figure 1:**
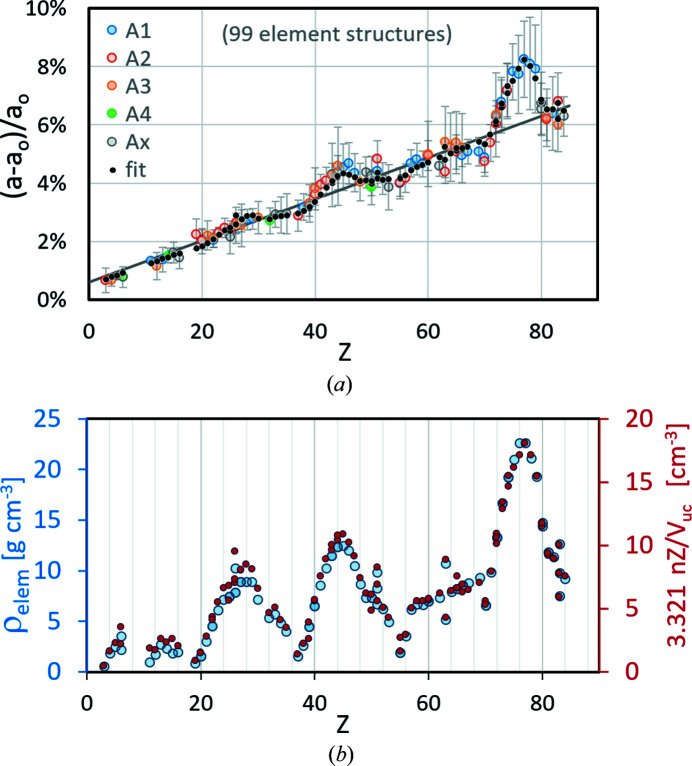
(*a*) The relative offset of the mean lattice parameter (*a*_*CALM*_ − *a*_0_)/*a*_0_ from simulated Kikuchi patterns of various single-element structures. The different colours of the hollow circles indicate the structure type: *A*1 = face-centred cubic (f.c.c.), *A*2 = body-centred cubic (b.c.c.), *A*3 = hexagonal close-packed (h.c.p.), *A*4 = diamond and *Ax* = other element structure types. The error bars refer to σ_*hkl*_. The predicted offsets considering both *Z* and the unit-cell volume *V*_uc_ are overlaid as black dots. (*b*) A plot to explain the significant deviations from the linear approach [dark-grey straight line in panel (*a*)], suggesting a correlation with the mass density ρ (left-hand axis) satisfyingly approximated by the right-hand axis (2 × 10^24^/*N*_A_)(*nZ*/*V*_uc_) (*N*_A_ is Avogadro’s number).

**Figure 2:**
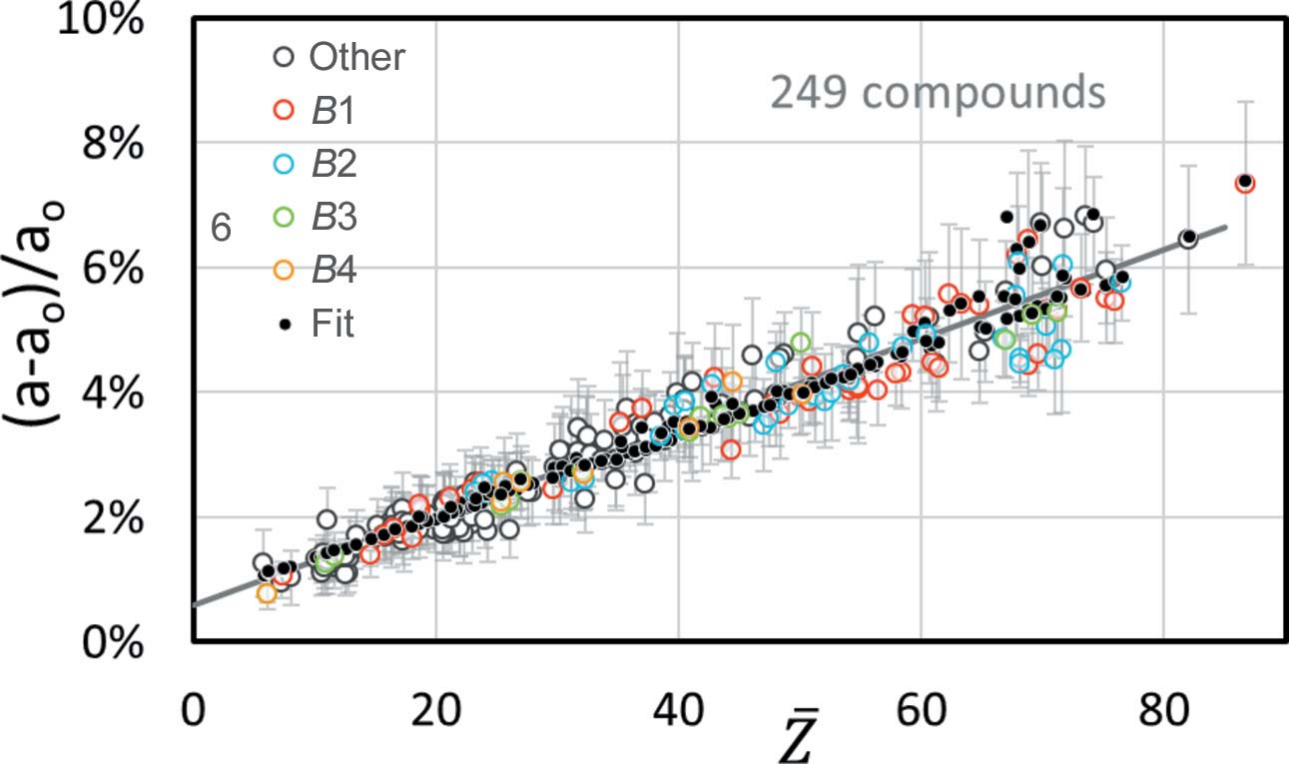
*CALM*-derived (hollow circles) and fitted (black dots) lattice parameter offsets for 249 different compounds. Both trace positions and the PC are known, and the error bars describe σ_*hkl*_ resulting from the band width distribution in each pattern. 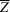 is derived by the mean of the products between the mass fraction *c*_*i*_ and atomic number *Z*_*i*_.

**Figure 3:**
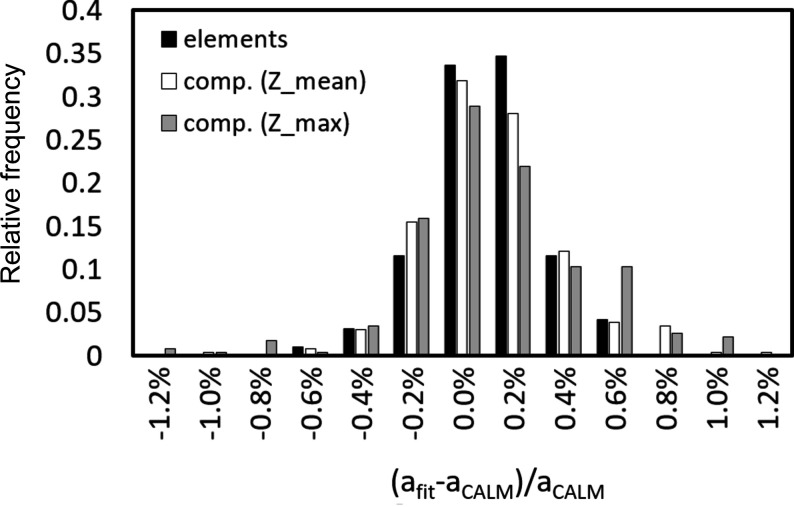
The relative frequency distribution of the deviations between the fitted lattice parameter calculated with equation (3) and the lattice parameter derived using *CALM*. Considered are 99 elements and 249 compounds. The application of *Z*_max_ instead of 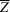 generates slightly higher predicted offsets, *cf*. the grey bar at 0.6%.

**Figure 4:**
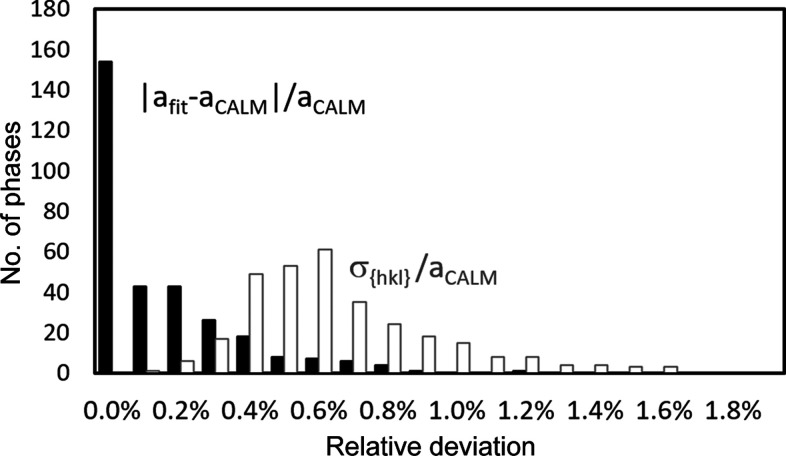
A comparison between the deviation of the relative lattice parameters derived using *CALM* and corrected with equation (3), and the band width-related standard deviation σ_*hkl*_.

**Figure 5:**
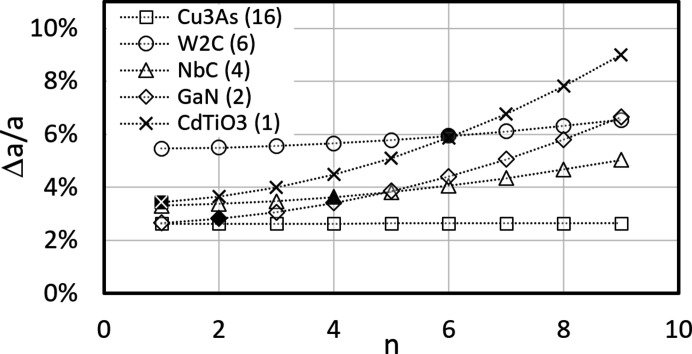
The variation in the offset Δ*a*/*a* as a function of *n*, the assumed number of formula units in the unit cell. The correct *n* is given in parentheses for each discussed phase. For Cu_3_As the use of *n* = 1 instead of 16 delivers no offset, for GaN the offset is ∼0.1%, and for NbC and W_2_C it is ∼0.3%.

**Figure 6:**
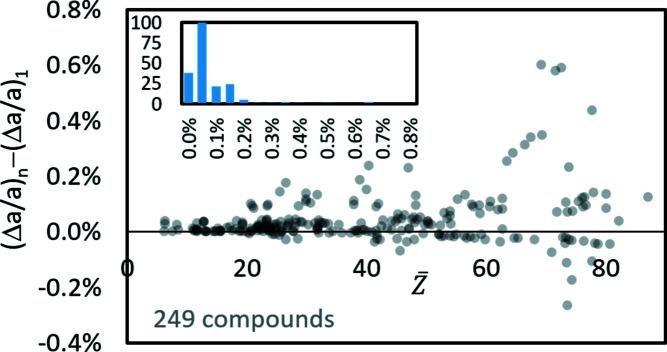
The difference between Δ*a*/*a* for *n* = 1 and the true *n*, displayed as semi-transparent discs. Additionally the histogram proves that, for most phases, this difference is lower than 0.1%. Higher deviations occur only for phases with higher 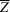 .

**Figure 7:**
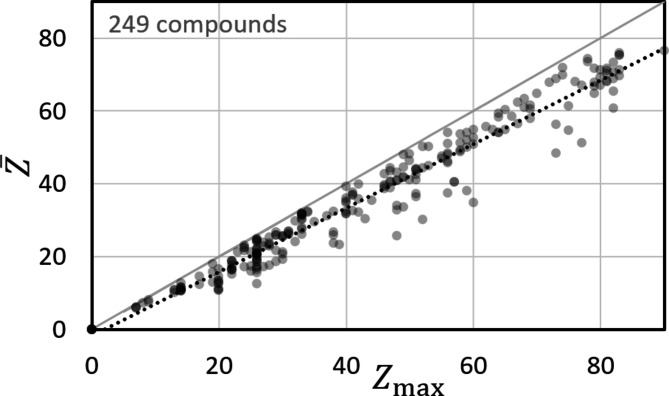
The deviation between 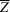 and *Z*_max_ for 249 compounds, displayed as semi-transparent dots. The solid light-grey line indicates the perfect match for elements ( 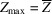 ). For compounds and alloys 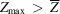 . The dotted line indicates a linear trend.

**Figure 8:**
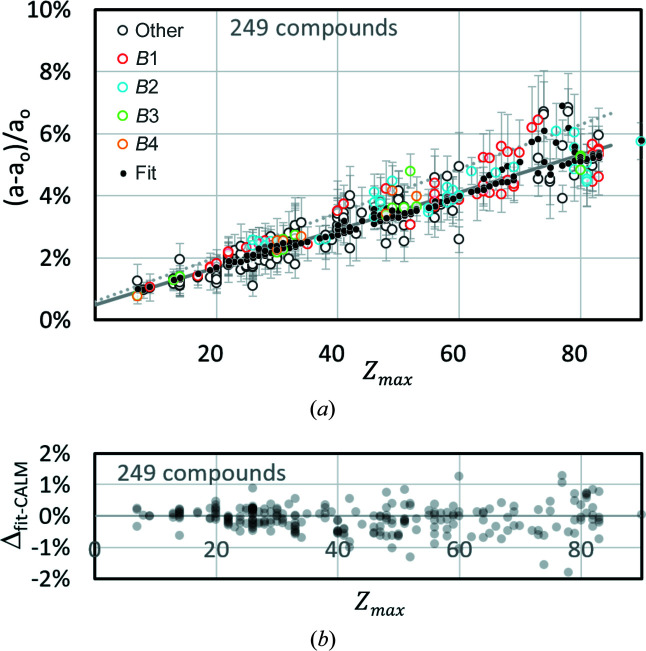
(*a*) The offset distribution as a function of *Z*_max_ for 249 compounds, displayed as hollow circles. For comparison, the light-grey dotted line indicates the offset level displayed in Fig. 2 ▸. The black dots represent the offset according to equation (3) with *Z* = *Z*_max_. (*b*) A plot highlighting the differences between the hollow circles and the respective black dots.

**Figure 9:**
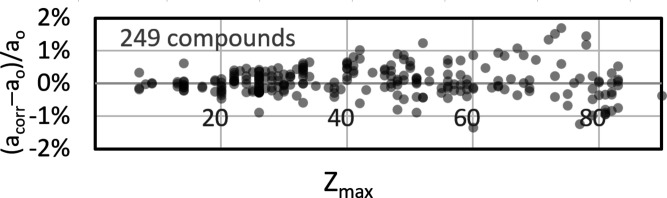
The relative deviation between the corrected lattice parameters derived using *CALM* and the first derivative, and the lattice parameter *a*_0_ used during Kikuchi pattern simulation.
